# The proteasome complex and the maintenance of pluripotency: sustain the fate by mopping up?

**DOI:** 10.1186/scrt413

**Published:** 2014-02-18

**Authors:** Friederike Schröter, James Adjaye

**Affiliations:** 1 Institute for Stem Cell Research and Regenerative Medicine, Medical Faculty, Heinrich Heine University, Moorenstraße 5, 40225 Düsseldorf, Germany; 2 Max-Planck-Institute for Molecular Genetics, Department for Vertebrate Genomics, Molecular Embryology and Aging Group, Ihnestraße 73, D-14195 Berlin, Germany

## Abstract

The proteasome is a multi-enzyme complex responsible for orchestrating protein quality control by degrading misfolded, damaged, abnormal and foreign proteins. Studies related to the association of the proteasomal system in the preservation of self-renewal in both human and mouse pluripotent cells are sparse, and therefore a clear indication of the emergence of a new and important field of research. Under specific conditions the standard proteasome switches to the newly synthesized immunoproteasome, a catalytically active protein chamber also involved in the regulation of protein homeostasis, cell signaling and gene expression. Herein we review recent data to help elucidate and highlight the pivotal role of the proteasome complex, constitutive as well as inducible, in the regulation of self-renewal, pluripotency and differentiation of both embryonic and induced pluripotent stem cells. The proteasome that is endowed with enhanced proteolytic activity maintains self-renewal by regulating gene expression. In addition to protein degradation, the proteasome activator PA28, compartments of the 19S regulatory particle and key members of the ubiquitin pathway dictate the fate of a pluripotent stem cell. We anticipate that our observations will stimulate active research in this new and emerging theme related to stem cell biology, disease and regenerative medicine.

## Introduction

### The ubiquitin–proteasome system

**Figure 1:**
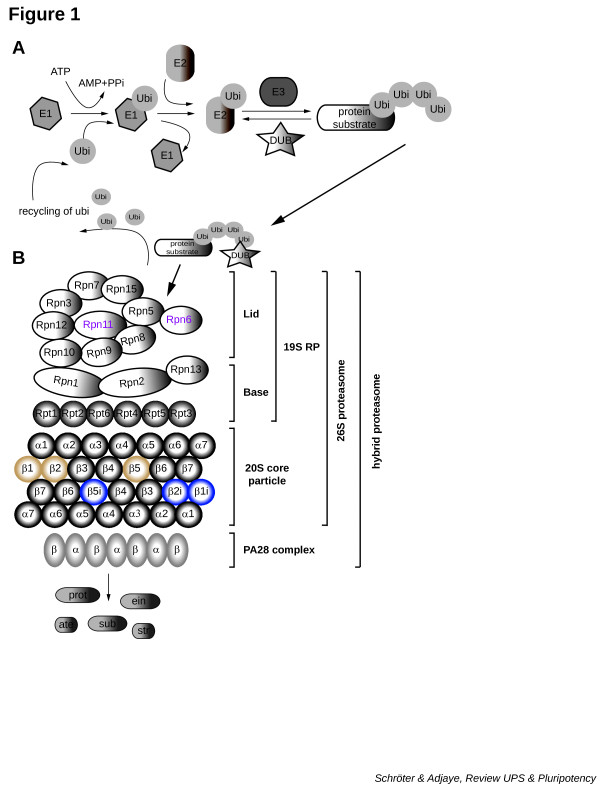
**The ubiquitin proteasome system. (A)** ATP-dependent activation of ubiquitin (ubi) by an E1 enzyme followed by ubi conjugation (E2) results in a high-energy E2-ubi thiol ester intermediate. The protein substrate binds via a defined recognition motif to a specific E3 ubi ligase – multiple repetition of this cycle provokes the synthesis of a poly-ubi chain to the protein substrate, ready for the degradation by the proteasome. For deeper insight, refer to [3]. **(B)** Two-dimensional diagram of the 26S and hybrid proteasome complex. Schematic presentation of the multi-enzyme complex including the 19S regulatory particle (RP) containing lid and base, the 20S core particle with the barrel-like structure of α7β7β7β7 and the proteasome activator complex PA28. 26S proteasome, 19S RP + 20S core; hybrid proteasome, 19S RP + 20S core + PA28. DUB, de-ubiquitinating enzyme; PPi, pyrophosphate; Rpn, non-ATPase subunit of 19S RP; Rpt, AAA-ATPase subunit of 19S RP.

The ubiquitin–proteasome system (UPS) is the proteolytic machinery operative in all eukaryotes to regulate basic cellular pathways such as cell cycle, signal transduction, transcription, protein turnover, response to oxidative stress and apoptosis [1,2]. Signals for post-translational modification are induced by covalent attachment of ubiquitin (ubi), clearly arranged by an ATP-requiring cascade of ubi-activating enzymes (E1; two distinct enzymes), ubi-conjugating enzymes (E2; there are dozens) and ubi ligases (E3; multiple E3s present) (Figure 1A) [3]. A single E1 interacts with all E2s, and distinct combinations of E2s and E3s enable substrate specificity and regulation [3]. Mono-ubi gives rise to processes associated with signal transduction and endocytosis, while poly-ubi marks the degradation of the substrate(s) by the proteasome in an ATP-dependent process (Figure 1) [4,5]. Ultimately the UPS controls intracellular protein homeostasis and quality during a cell’s life and death, and plays major roles in both health and disease – for example, Alzheimer’s disease (neuro-degenerative), transient ischemia (cardiac dysfunction) and Sjorgen’s syndrome (autoimmune) (reviewed in [6]).

The central core of the proteasome, the 20S complex, has a barrel-like structure composed of four rings that consist of seven subunits (α7β7β7β7) (Figure 1B). Only three of the seven beta subunits harbor the active threonine site (Thr1) at the N-termini [7], serving the nucleophile for proteolytic hydrolysis: the *PSMB6*-encoded β1 subunits catalyze caspase-like activity; the *PSMB7*-encoded β2 subunits catalyze trypsin-like activity; and the *PSMB5*-encoded β5 subunits catalyze chymotrypsin-like (Chy-L) activity (see Figure 1B, tan-colored subunits; and Table 1) [2,8]. The 20S complex requires the 19S regulatory particle (RP) to degrade poly-ubi substrates and is called the 26S (single cap) or 30S (double cap) proteasome complex (Figure 1B) [2,9]. The 20S core complex functions independently of ATP and is unable to degrade poly-ubi substrates, unlike the 26S/30S proteasome [10].

**Table 1:** Overview of the specific proteasome subunits that influence the pluripotent and differentiated state

**Category**	**Systemic nomenclature**	**HUGO**	**Function**	**References**
19S lid	Rpn6	PSMD11	Stabilize interaction between 19S RP and 20S core	[2,30,32,35,36]
	Rpn11	PSMD14	De-ubiquitinating enzyme	
	Rpn12	PSMD8		
19S base	Rpt3	PSMC4	ATPase, gate-opening	[2,30]
20S core particle	β1	PSMB6	Caspase-like	[2,8,18]
	β2	PSMB7	Trypsin-like	
	β5	PSMB5	Chymotrypsin-like	
	β1i	PSMB9	Chymotrypsin-like	
	β2i	PSMB10	Trypsin-like	
	β5i	PSMB8	Chymotrypsin-like	
PA28 complex	(PA28α)	PSME1	Proteasome activator	[2,13,25]
	(PA28β)	PSME2		

Explanation of the different nomenclature of the proteasome subunits as well as their functions that are important for the pluripotent and differentiated cell fate. HUGO, Human Genome Organization; RP, regulatory particle; Rpn, non-ATPase subunit of 19S RP; Rpt, AAA-ATPase subunit of 19S RP.

The 19S RP itself is formed by the base and the lid, containing 18 subunits separated into 13 non-ATPase (Rpn) subunits and six AAA-ATPase (Rpt) subunits (Figure 1B) [11]. The functions of the RP include: capturing the poly-ubi proteins via PSMD4/RPN10 and ADRM1/RPN13 (lid); de-ubiquitinating these captured substrates (lid; mainly by the de-ubiquitinating enzymes (DUBs)); and promoting the unfolding of these substrates, completed with the opening of the α-rings for the entry into the central chamber (base; Rpt1 to Rpt6) [2,11,12]. The 20S core particle can be activated by the 19S RP and also by the proteasome activator PA28, which is a homoheptameric complex consisting of PA28α and PA28β subunits (Figure 1B) [2]. The C-termini of PA28 intercalate into the intersubunit pocket between adjacent α subunits and controls, and also stabilize the gate opening especially during immune response [2,13]. The contacts with inflammatory mediators provoke the substitution of the constitutive to the inducible catalytically active subunits, converting the proteasome complex into the immunoproteasome (iP). The integration of the inducible subunits PSMB9/β1i, PSMB10/β2i and PSMB8/β5i in the 20S chamber (Figure 1B, blue-colored subunits) alters peptidase activities, thus resulting in a higher efficiency in the generation of selected major histocompatibility complex class I epitopes [14-17]. The variation of the epitopes generated by the inducible proteasome complex originate from the enhanced cleavage after basic and hydrophobic residues (trypsin-like activity and Chy-L activities), while the degradation after acidic amino acids is reduced (caspase-like activity; see Table 1) [18,19].

## Review

The molecular signature of human embryonic stem cells (hESCs) was first described by Sato and colleagues [20], identifying about 900 genes including *PSMB8/β5i* among the top 10. Subsequently, several studies also identified a set of genes encoding the UPS [21-23], again further evidence supporting the role for the proteasome machinery in the maintenance of pluripotency. Accordingly, proteomic analysis of three distinct hESC lines (H2, H3, H5) identified components of the UPS, especially an enrichment of distinct proteasomal subunits [22]. Remarkably, the subunits that do not possess the catalytically active sites were over-represented in these analyses [21-23]. On the contrary, unsolved mystery exists and there are several open questions: what is the relevance of the interplay of the different subunits of the multi-enzyme; is the functionality of the UPS imparted by the catalytically active subunits; do pluripotent stem cells need more proteasomal activity; and would inhibition of the proteasome affect the maintenance and self-renewal of pluripotent cells? Answers to some of these questions are given in the subsequent sections of this review.

## The proteasome complex in murine pluripotent stem cells

### Removal of damaged proteins in pluripotent cells by the proteasome

Evidence in support of the central role of the UPS in the maintenance and induction of pluripotency in mouse embryonic stem cells (mESCs) and mouse somatic cells has been provided and is reviewed by Naujokat and Saric [24]. Interestingly, oxidatively modified proteins such as carbonylated proteins and advanced glycation end products accumulate in mESCs as a consequence of reactive oxygen species [25]. These modified proteins are enriched in ubi conjugates [26], which naturally results in proteasomal degradation.

One has to note that the protein level in the cell *per se* remains unaltered, whilst the level of oxidatively damaged proteins diminishes during the differentiation of murine stem cells [13,25]. The reduction in the amount of oxidative-modified proteins is a result of the enhanced activity of the 20S and not that of the ATP-dependent 26S proteasome [25]. The *de novo* synthesized iP is processed during transient adaption to oxidative stress [27,28], thus leading to the assumption that the inducible subunits in mESCs should be expressed in order to mediate the cleavage of the observed damaged proteins. On the contrary, elevated levels of Psmb8/β5i as well as of the PA28α/PA28β subunits are detected upon embryoid body-mediated differentiation [13].

**Figure 2:**
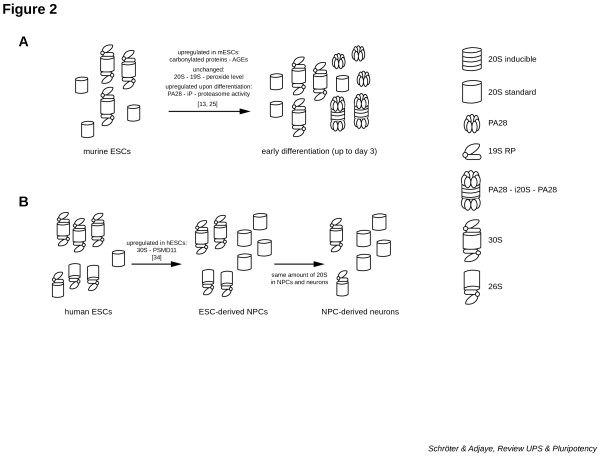
**Scheme of the distinct proteasome-types present in murine and human pluripotent and differentiated cells. (A)** An abundance of 30S proteasome is presented in mouse embryonic stem cells (mESCs) compared with 3-day differentiated ESCs. The PA28 complex is induced upon differentiation and anchored to the inducible proteasome complex*.***(B)** Downregulated expression of the 30S complex upon neural progenitor cells (NPCs) and neuronal differentiation is found. In contrast to the human ESCs (hESCs), the neural cells have more free 20S complex. AGE, advanced glycation end product; iP, immunoproteasome; RP, regulatory particle.

Furthermore, no significant changes in the expression levels of the proteasome maturation protein and a constant level of both 20S core and 19S RP are observed in pluripotent cells compared with their differentiated counterparts [13,25]. The unaltered level of the 26S proteasome complex coupled to the drastically increased levels of the 20S complex with enhanced proteasomal activity and also increased levels of PA28 during the switch from a pluripotent to a somatic cell suggest that the ATP-ubi-independent PA28–iP–PA28 complex (double-cap PA28 on iP-20S core; Figure 2A) as well as the ATP-ubi-dependent hybrid proteasome (PA28–20S–19S; Figure 1B) in combination with the inducible catalytically active subunits are predominantly responsible for the reduction in the amounts of oxidatively modified proteins [13,25].

In murine somatic cells such as skin fibroblasts, murine embryonic fibroblasts (MEFs) as well as liver and brain tissue, the level of protein oxidation depends on degradation by iP together with the appropriately assembled hybrid and PA28 proteasome [27-29]. Evidence provided so far implies that iP and PA28 play major roles in the degradation of oxidized proteins in somatic cells as well as the process of differentiation, but not in pluripotent cells. All in all, oxidatively damaged proteins in part govern the steady state of pluripotency in mESCs but are mopped up by the proteasome during the process of specified decisions to exit self-renewal.

### Influence of the proteasome in gene transcription on pluripotent cells

The current view is that the proteasome is involved in the removal of damaged proteins upon differentiation, but is it also involved in maintaining gene regulatory networks needed to sustain pluripotency in mESCs? Pluripotent cells have the ability to give rise to all cell types found in the body, and this therefore assumes a tight control of self-renewal and pluripotency. This strict program is accompanied by a highly accessible chromatin state without any influence from tissue-specific activators in mESCs [30]. Remarkably the inhibition of proteolytic activity or the depletion of distinct proteasomal subunits in mESCs results in enhanced binding of specific transcription factors and RNA polymerase II itself, followed by the activation of cryptic promoters, which are epigenetically silenced and normally inactive [30]. In contrast to the cryptic promoter, proteolysis by the 20S proteasome at active promoters maintains transcriptional elongation by displacing RNA polymerase II [30]. An implication of this observation is that the proteasomal degradation in mESCs operates at tissue-specific gene loci to inhibit the binding of tissue-specific transcription factors and/or RNA polymerase II, thus repressing transcription of their target genes. This further implies that the proteasome acts as a transcriptional silencer in mESCs in order to maintain pluripotency [30].

Similar to the proteolytic activity, the 19S RP regulates gene expression by interfering with chromatin modifications. In mESCs, the Psmd8/Rpn12 subunit (lid) controls the assembly of specific and nonspecific pre-initiation complexes, a large complex of proteins necessary for transcription, but only in the presence of the Psmc4/Rpt3 subunit (base) [30]. These findings would imply that the proteasome complex itself acts on specific regulatory regions in mESCs to prevent aberrant transcriptional initiation that would otherwise initiate the exit from pluripotency.

### Control of ubiquitin ligase and de-ubiquitinating enzyme in murine pluripotent stem cells

The UPS is also involved in cell cycle control. One of the key mechanisms underlying cell cycle control is the ubi-mediated proteolysis of regulatory molecules (for example, cyclin, cyclin-dependent kinases and cyclin-dependent kinase inhibitors): two multi-protein E3 ubi ligase complexes – the Skp1-cullin-F-box protein complex and the anaphase-promoting complex – are involved in cell cycle regulation (reviewed in [31]).

The F-box protein Fbw7/Fbxw7 influences through its ubi ligase activity the degradation of crucial cell cycle regulators such as c-Myc, Jun, Cyclin-E and Notch [31]. Upon binding to its specific target, umpteen cell type-dependent networks of proteins are orchestrated by the E3 Fbxw7 [31]. The expression of Fbxw7 is similar in mESCs (and murine induced pluripotent stem cells (iPSCs)) compared with MEFs [32]. Interestingly, during the differentiation of mESCs, Fbxw7 is upregulated while c-Myc, an essential regulator of self-renewal and pluripotency, is downregulated [32]. Furthermore, depletion of Fbxw7 in mESCs induces elevated expression of c-Myc, Oct4, Nanog and Sox2 upon early differentiation, and reprogramming Fbxw7-silenced MEFs induces enhanced efficiency in the derivation of iPSCs [32].

Not only does an E3 ubi ligase regulate pluripotency, but also a member of the lid, the DUB Psmd14/Rpn11, seems to be a crucial factor essential for maintaining pluripotency [32]. Psmd14/Rpn11 is expressed in mESCs and is downregulated upon differentiation [32]. Depletion of the lid subunit in MEFs results in the opposite effect observed with the knockdown of Fbxw7; that is, suppression of cellular reprogramming [32]. Interestingly, the abolition of DUB activity results in the exit of self-renewal and pluripotency. In addition, the overexpression of Psmd14/Rpn11 in mESCs antagonized the differentiation and supported the pluripotent state [32]. This study once again shows that components of the USP are essential for cellular reprogramming and the regulation of the core pluripotency machinery. Remarkably the proteasomal subunits that regulate self-renewal and pluripotency are ubi ligase, responsible for ubi tagging, and de-ubiquitinase, responsible for the removal of ubi – this is an example of how both sides of the coin are crucial for maintaining self-renewal and pluripotency in murine embryonic stem cells and iPSCs.

### Post-translational modifications of pluripotency-associated transcription factors

Buckley and colleagues inhibited proteasome activity employing MG132 (concentration and duration not specified) for the analysis of proteasome-dependent protein turnover in mESCs and iPSCs [32]. Interestingly, Oct4, Sox2, Nanog and c-Myc, as well as Dax1, Rex1, Dnmt3l and Msh6, are conjugated by ubi [32,33] and thus are regulated by the UPS in these cells. This is strong evidence for the inclusion of the UPS as a pluripotency-associated regulator. It is important to bear in mind that ubi-mediated proteasomal degradation is not exclusively responsible for post-translational modifications in mESCs. Buckley and colleagues found an overlap of phosphorylated and ubiquitinated proteins (289 in number), and demonstrated that a considerable number are associated with pluripotency [32]. Additionally, the interplay between post-translational modifications and ubi-mediated degradation seems to be crucial for maintaining self-renewal and pluripotency.

## The proteasome complex in human pluripotent stem cells

Several subunits of the lid are interacting proteins of the DUB PSMD14/RPN11, including, for example, PSMD11/RPN6 (Table 1). Similar to the murine DUB Psmd14/Rpn11, the non-ATPase subunit PSMD11/RPN6 plays a key role in human pluripotent cells [34]. Generally the lid subunit PSMD11/RPN6 functions to stabilize the interaction of the 19S RP to the 20S core via the α2 subunit [35,36]. PSMD11/RPN6 is expressed at high levels in both embryonic stem cells and iPSCs. Differentiation of hESCs to neural progenitor cells (NPCs) and to mature neurons results in a downregulated expression of PSMD11/RPN6 [34]. This depletion of PSMD11/RPN6 results in reduced proteasomal activity and consequently a reduction in the amount of assembled proteasome complex (Figure 2B). Downregulated expression of PSMD11/RPN6 during differentiation is accompanied by a decrease in hydrolytic activity of the proteasome complex. This observation is proof that PSMD11/RPN6 is essential for preserving the activity of the proteasome.

Analyses of the synthesized and functional proteasome complex in hESCs compared with derived NPCs and neurons revealed a higher amount of 30S proteasome (double-cap 19S) and less 20S complex (Figure 2B). Commonly it seems that there is reduced proteasomal activity in all hESC-derived cells such as trophoblasts, as well as somatic cells such as human cortical/hippocampal astrocytes, fibroblasts and HEK293T [34]. In general, the UPS plays a pivotal role in neurons, especially in synaptic transmission [37], so there is no plausible explanation as to why NPCs or mature neurons should have less functional proteasome complexes compared with pluripotent cells.

hESCs probably also possess high levels of damaged proteins, as demonstrated in mESCs [13,25]. Remarkably, human pluripotent cells possess less oxidatively modified proteins compared with human neonatal foreskin fibroblast (HFF)-1, embryonic stem cell-derived and iPSC-derived fibroblast-like cells [38]; the opposite is true for mESCs [13,25]. In addition, the authors show a higher resistance of pluripotent cells to oxidative stress [38]. Additionally, the increased amount of available 20S core upon NPC and neuronal differentiation of hESCs deflects the question of whether the PA28 complex also plays an essential role in this process. Hernebring and coworkers demonstrated that the proteasome activator is responsible for the elevated proteolytic activity in differentiated mESCs [13]. The possibility exists that the free 20S complex, standard or inducible, can be tagged by the PA28 particle; however, this process induces an elevated level of proteolytic activity [13,25], and therefore would suggest the presence of the inducible proteasome complex [27,28].

### Immunoproteasome in pluripotent and differentiated cells

The iP is a subtle modification of the proteasome composition and is mainly associated with antigen processing, protein homeostasis and oxidative stress responses [28,39]. To investigate the role of iP in maintaining pluripotency, Atkinson and colleagues observed the loss of Chy-L activity during embryoid body-based differentiation of hESCs [40]. This observation is in line with the decline of the 26S activity upon differentiation in hESCs [34] and contrary to the boost of the 26S activity upon mESCs-derived differentiation [13,25]. The focus of this publication is on both the constitutive and inducible proteasome complex [40].

Chy-L activity is brought about by PSMB5/β5, PSMB9/β1i and PSMB8/β5i (Table 1) [8,14,41]. Remarkably, the mRNA expression levels of the constitutive beta subunits (PSMB6/β1, PSMB7/β2) were downregulated whilst the levels of the PSMB5/β5 subunits remained unchanged upon embryoid body-based differentiation of hESCs [40]. In contrast to the transcriptional level, the protein level of the catalytically active constitutive subunits remained unaltered [40]. A loss of inducible PSMB9/β1i and PSMB8/β5i expression at both the RNA and protein levels was observed after 16 days of embryoid body-mediated differentiation of hESCs [40], which is further evidence in support of the observed reduced Chy-L activity upon the loss of pluripotency. Is it therefore possible that the observed enhanced proteasomal activity in pluripotent cells is detected simply because of the iP? The studies described in [32,34] did not investigate the role of the iP complex in maintaining pluripotency. The iP itself, beyond its function of generating major histocompatibility complex class I epitopes, has a higher protein turnover ratio [28], a shorter half-life [17] and cleans up after inflammation [28] compared with the constitutive proteasome complex. Further experiments are warranted in order to elucidate signaling mechanisms beyond the (inducible) proteasomal activity during exit of self-renewal.

## Inhibition of proteasome activity affects the induction, maintenance and exit of pluripotency

Recent microarray-based transcriptome analysis of RNAi-based depletion of OCT4 function in the hESC H1 cell line revealed the regulation of 18 genes associated with the proteasome pathway [23]. Altered proteasome activity in ESCs stimulated with distinct proteasome inhibitors elicits different effects as summarized in Table 2. Remarkably, inhibition of the proteasome using MG132, a reversible and cell-permeable inhibitor, affects only pluripotent stem cells and not somatic cells such as HFF and fibroblast derived from HFF-iPSCs (Table 2) [21,34,40]. Short-term treatment (~20 minutes up to 10 hours) with high concentrations of distinct inhibitors (20 μM MG132/8 to 10 hours [30]; 25 μM MG132, 30 μM PI-I, 10 μM lactacystin/20 minutes each [34]) did not alter cell viability, morphology or self-renewal. Interestingly, however, a low dose of MG132 stimulation in MEFs inhibited cellular reprogramming (Table 2) [32]. In contrast, inhibition of proteasomal activity in pluripotent cells always led to a downregulated expression of pluripotency-associated genes such as *OCT4*, *NANOG*, *c-MYC*, *SOX2*, *SSEA3*, *TRA-1-81* and *TRA-1-60*, and therefore loss of self-renewal with concomitant activated expression of differentiation markers such as *FGF5* and *GATA4* (Table 2) [21,34,40]. Moreover, an accumulation of the ubi-tagging on Oct4, Nanog, c-Myc and p53 has been described [32].

**Table 2:** Summary of proteasome inhibitors and their impact on pluripotency

**Cell type**	**Concentration, inhibitor/time**	**Effect**	**Pluripotency marker**	**Reference**
HFF/iPSC-derived fibroblast	250, 500 and 1,000 nM MG132/40 hours	No morphological change	Nondetectable	[21]
Fibroblasts	2 μM UK101/PD957	Complete inhibition of reprogramming	Nondetectable	[40]
MEFs	Low doses of MG132	Complete inhibition of reprogramming	Nondetectable	[32]
hESC-derived fibroblasts	250, 500 and 1,000 nM MG132/40 hours	No morphological change	Nondetectable	[21]
hESCs	125 nM MG132/40 hours	No effect	Significant downregulation of *OCT4*, *SOX2*, *NANOG* and *TRA-1-60*	[21]
	250 nM MG132/40 hours	Large patches of differentiated areas		
	500 nM MG132/40 hours	Only differentiated cells		
	1,000 nM MG132/40 hours	Detachment of undifferentiated cells		
hESCs	62.5 nM MG132/24 hours	Downregulation of pluripotent markers and modified levels of specific germ-layer markers (upregulation of FGF5 and GATA4)	Significant downregulation of *OCT4*, *NANOG*, *SOX2* and *ZFP42*	[34]
hESCs	2 μM UK101/PD957 for 4 days	Pluripotency diminished, less alkaline phosphatase positive colonies, upregulation of FGF5 and GATA4	Significant downregulation of *TRA-1-81* and *SSEA3*	[40]

Application of distinct proteasome inhibitors on different cell types, the effect of inhibition and the influence on pluripotency-associated transcription factors. hESC, human embryonic stem cell; HFF, human neonatal foreskin fibroblast; iPSC, induced pluripotent stem cell; MEF, murine embryonic fibroblast.

The use of specific inhibitors of immunoproteasomal activity, UK101 (PSMB9/β1i) and PK957 (PSMB8/β5i), reduced the efficiency of cellular reprogramming, induced an exit of self-renewal and activated expression of somatic markers such as *FGF5* and *GATA4* (Table 2) [40]. This observation is evidence that the iP complex is essential for the induction of pluripotency in somatic cells.

Depending on the concentration of proteasome inhibitor used, one can influence cell fate – high doses lead to the exit of self-renewal [21,34,40] and inhibition of cellular reprogramming [32,40]. The entire ATP-dependent proteolytic machinery, including the inducible complex, seems to be a regulator of self-renewal capability.

## Conclusions

The proteasome complex influences the induction, maintenance and exit of self-renewal and pluripotency in both mouse and human. First, together with the PA28 complex, the proteasome complex removes the high amount of oxidatively damaged proteins upon differentiation [13,25]. Second, the complex acts as a gene silencer in mESCs [30]. Third, the complex regulates the pluripotency-associated cell cycle via E3 ubi ligase and DUB [32]. Fourth, the complex modulates pluripotency via post-translational modifications such as ubi-tagging of the core pluripotency-associated transcription factors, for example Oct4, Nanog and c-Myc [13,32]. Fifth, proteasome inhibition induces an exit of self-renewal [21,34,40] and the inhibition of the induction of pluripotency in somatic cells [32,40]. It is now evident that the UPS plays a pivotal role in maintaining pluripotency as well as supporting the mopping up of damaged proteins during differentiation.

The iP is so far accepted to fulfill almost the same functions as the standard proteasome. These functions include protein homeostasis, cell proliferation and differentiation, transcription and major histocompatibility complex class I signaling. To date, the functional relevance of the inducible proteasome in the maintenance of pluripotency and self-renewal in human embryonic and induced pluripotent stem cells or in the induction of pluripotency in somatic cells remains unexplored. Notably, the inducible subunits PSMB8/β5i and PSMB9/β1i are also expressed in human placenta [39]. Further experiments are warranted to enable clarification of the pivotal role played by this fascinating inducible multi-enzyme protein complex in the induction, maintenance and loss of pluripotency. The necessity for the degrading machinery in maintaining self-renewal and pluripotent is evident [21,34,40]. The importance of the proteolytic machinery lies in the rapid modification of the cell cycle, regulation of transcription and control of protein damage (oxidatively and/or carbonylated) to sustain the proliferation capacity of pluripotent cells.

As embryonic stem cells and iPSCs have the unique ability to self-renew and are pluripotent, they are capable of differentiating into cell types representative of the three embryonic germ layers: mesoderm, endoderm and ectoderm [42]. Murine embryonic stem cells and iPSCs maintain pluripotency by virtue of a gene regulatory network based on the leukemia inhibitory factor and canonical Wnt pathway [43], whereas in human this depends on the fibroblast growth factor and transforming growth factor beta/NODAL/ACTIVIN-signaling pathways [44,45]. It is well known that the UPS has a bearing on these signaling pathways beside its regulatory functions (leukemia inhibitory factor [46], transforming growth factor beta [47], β-catenin/Wnt [48]). An in-depth understanding of these signaling pathways and the involvement of the UPS in maintaining self-renewal is urgently needed.

## Abbreviations

Chy-L: Chymotrypsin-like activity; DUB: De-ubiquitinating enzyme; hESC: Human embryonic stem cell; HFF: Human neonatal foreskin fibroblast; iP: Immunoproteasome; iPSC: Induced pluripotent stem cell; MEF: Murine embryonic fibroblast; mESC: Mouse embryonic stem cell; NPC: Neural progenitor cell; RP: Regulatory particle; Rpn: Non-ATPase subunit of 19S RP; Rpt: AAA-ATPase subunit of 19S RP; PSMB: Proteasome beta subunit; PSMD: Proteasome delta subunit; ubi: Ubiquitin; UPS: Ubiquitin–proteasome system.

## Competing interests

The authors declare that they have no competing interests.

## References

[B1] Kloetzel PM Soza A Stohwasser R The role of the proteasome system and the proteasome activator PA28 complex in the cellular immune response Biol Chem 1999 380 293 297 10223331 10.1515/BC.1999.040

[B2] Tanaka K The proteasome: overview of structure and functions Proc Jpn Acad Ser B Phys Biol Sci 2009 85 12 36 10.2183/pjab.85.12 19145068 PMC3524306

[B3] Glickman MH Ciechanover A The ubiquitin–proteasome proteolytic pathway: destruction for the sake of construction Physiol Rev 2002 82 373 428 11917093 10.1152/physrev.00027.2001

[B4] Mukhopadhyay D Riezman H Proteasome-independent functions of ubiquitin in endocytosis and signaling Science 2007 315 201 205 10.1126/science.1127085 17218518

[B5] Seifert U Kruger E Remodelling of the ubiquitin-proteasome system in response to interferons Biochem Soc Trans 2008 36 879 884 10.1042/BST0360879 18793155

[B6] Dahlmann B Role of proteasomes in disease BMC Biochem 2007 8 S3 10.1186/1471-2091-8-S1-S3 18047740 PMC2106367

[B7] Fenteany G Standaert RF Lane WS Choi S Corey EJ Schreiber SL Inhibition of proteasome activities and subunit-specific amino-terminal threonine modification by lactacystin Science 1995 268 726 731 10.1126/science.7732382 7732382

[B8] Orlowski M Wilk S Catalytic activities of the 20 S proteasome, a multicatalytic proteinase complex Arch Biochem Biophys 2000 383 1 16 10.1006/abbi.2000.2036 11097171

[B9] Kloetzel PM The proteasome and MHC class I antigen processing Biochim Biophys Acta 2004 1695 225 233 10.1016/j.bbamcr.2004.10.004 15571818

[B10] Kisselev AF Goldberg AL Monitoring activity and inhibition of 26S proteasomes with fluorogenic peptide substrates Methods Enzymol 2005 398 364 378 16275343 10.1016/S0076-6879(05)98030-0

[B11] da Fonseca PC He J Morris EP Molecular model of the human 26S proteasome Mol Cell 2012 46 54 66 10.1016/j.molcel.2012.03.026 22500737

[B12] Smith DM Chang SC Park S Finley D Cheng Y Goldberg AL Docking of the proteasomal ATPases' carboxyl termini in the 20S proteasome's alpha ring opens the gate for substrate entry Mol Cell 2007 27 731 744 10.1016/j.molcel.2007.06.033 17803938 PMC2083707

[B13] Hernebring M Fredriksson A Liljevald M Cvijovic M Norrman K Wiseman J Semb H Nyström T Removal of damaged proteins during ES cell fate specification requires the proteasome activator PA28 Sci Rep 2013 3 1381 23459332 10.1038/srep01381 PMC3587881

[B14] Boes B Hengel H Ruppert T Multhaup G Koszinowski UH Kloetzel PM Interferon gamma stimulation modulates the proteolytic activity and cleavage site preference of 20S mouse proteasomes J Exp Med 1994 179 901 909 10.1084/jem.179.3.901 8113682 PMC2191397

[B15] Sijts AJ Ruppert T Rehermann B Schmidt M Koszinowski U Kloetzel PM Efficient generation of a hepatitis B virus cytotoxic T lymphocyte epitope requires the structural features of immunoproteasomes J Exp Med 2000 191 503 514 10.1084/jem.191.3.503 10662796 PMC2195811

[B16] Kruger E Kuckelkorn U Sijts A Kloetzel PM The components of the proteasome system and their role in MHC class I antigen processing Rev Physiol Biochem Pharmacol 2003 148 81 104 10.1007/s10254-003-0010-4 12687403

[B17] Strehl B Seifert U Kruger E Heink S Kuckelkorn U Kloetzel PM Interferon-gamma, the functional plasticity of the ubiquitin-proteasome system, and MHC class I antigen processing Immunol Rev 2005 207 19 30 10.1111/j.0105-2896.2005.00308.x 16181324

[B18] Gaczynska M Rock KL Goldberg AL Role of proteasomes in antigen presentation Enzyme Protein 1993 47 354 369 7697133 10.1159/000468693

[B19] Gaczynska M Rock KL Goldberg AL Gamma-interferon and expression of MHC genes regulate peptide hydrolysis by proteasomes Nature 1993 365 264 267 10.1038/365264a0 8396732

[B20] Sato N Sanjuan IM Heke M Uchida M Naef F Brivanlou AH Molecular signature of human embryonic stem cells and its comparison with the mouse Dev Biol 2003 260 404 413 10.1016/S0012-1606(03)00256-2 12921741

[B21] Assou S Cerecedo D Tondeur S Pantesco V Hovatta O Klein B Hamamah S De Vos J A gene expression signature shared by human mature oocytes and embryonic stem cells BMC Genomics 2009 10 10 10.1186/1471-2164-10-10 19128516 PMC2628676

[B22] Baharvand H Hajheidari M Ashtiani SK Salekdeh GH Proteomic signature of human embryonic stem cells Proteomics 2006 6 3544 3549 10.1002/pmic.200500844 16758447

[B23] Babaie Y Herwig R Greber B Brink TC Wruck W Groth D Lehrach H Burdon T Adjaye J Analysis of Oct4-dependent transcriptional networks regulating self-renewal and pluripotency in human embryonic stem cells Stem Cells 2007 25 500 510 10.1634/stemcells.2006-0426 17068183

[B24] Naujokat C Saric T Concise review: role and function of the ubiquitin–proteasome system in mammalian stem and progenitor cells Stem Cells 2007 25 2408 2418 10.1634/stemcells.2007-0255 17641241

[B25] Hernebring M Brolen G Aguilaniu H Semb H Nystrom T Elimination of damaged proteins during differentiation of embryonic stem cells Proc Natl Acad Sci U S A 2006 103 7700 7705 10.1073/pnas.0510944103 16672370 PMC1472508

[B26] Dudek EJ Shang F Valverde P Liu Q Hobbs M Taylor A Selectivity of the ubiquitin pathway for oxidatively modified proteins: relevance to protein precipitation diseases FASEB J 2005 19 1707 1709 16099947 10.1096/fj.05-4049fje

[B27] Pickering AM Koop AL Teoh CY Ermak G Grune T Davies KJ The immunoproteasome, the 20S proteasome and the PA28alphabeta proteasome regulator are oxidative-stress-adaptive proteolytic complexes Biochem J 2010 432 585 594 10.1042/BJ20100878 20919990 PMC3133595

[B28] Seifert U Bialy LP Ebstein F Bech-Otschir D Voigt A Schroter F Prozorovski T Lange N Steffen J Rieger M Kuckelkorn U Aktas O Kloetzel PM Krüger E Immunoproteasomes preserve protein homeostasis upon interferon-induced oxidative stress Cell 2010 142 613 624 10.1016/j.cell.2010.07.036 20723761

[B29] Ebstein F Voigt A Lange N Warnatsch A Schroter F Prozorovski T Kuckelkorn U Aktas O Seifert U Kloetzel PM Krüger E Immunoproteasomes are important for proteostasis in immune responses Cell 2013 152 935 937 10.1016/j.cell.2013.02.018 23452842

[B30] Szutorisz H Georgiou A Tora L Dillon N The proteasome restricts permissive transcription at tissue-specific gene loci in embryonic stem cells Cell 2006 127 1375 1388 10.1016/j.cell.2006.10.045 17190601

[B31] Tu Y Chen C Pan J Xu J Zhou ZG Wang CY The ubiquitin proteasome pathway (UPP) in the regulation of cell cycle control and DNA damage repair and its implication in tumorigenesis Int J Clin Exp Pathol 2012 5 726 738 23071855 PMC3466981

[B32] Buckley SM Strikoudis A Apostolou E Loizou E Moran-Crusio K Farnsworth CL Koller AA Dasgupta R Silva JC Stadtfeld M Hochedlinger K Chen EI Aifantis I Aranda-Org Regulation of pluripotency and cellular reprogramming by the ubiquitin–proteasome system Cell Stem Cell 2012 11 783 798 10.1016/j.stem.2012.09.011 23103054 PMC3549668

[B33] Park JA Kim YE Ha YH Kwon HJ Lee Y High sensitivity of embryonic stem cells to proteasome inhibitors correlates with low expression of heat shock protein and decrease of pluripotent cell marker expression BMB Rep 2012 45 299 304 10.5483/BMBRep.2012.45.5.299 22617454

[B34] Vilchez D Boyer L Morantte I Lutz M Merkwirth C Joyce D Spencer B Page L Masliah E Berggren WT Gage FH Dillin A Increased proteasome activity in human embryonic stem cells is regulated by PSMD11 Nature 2012 489 304 308 10.1038/nature11468 22972301 PMC5215918

[B35] Lander GC Estrin E Matyskiela ME Bashore C Nogales E Martin A Complete subunit architecture of the proteasome regulatory particle Nature 2012 482 186 191 22237024 10.1038/nature10774 PMC3285539

[B36] Pathare GR Nagy I Bohn S Unverdorben P Hubert A Korner R Nickell S Lasker K Sali A Tamura T Nishioka T Förster F Baumeister W Bracher A The proteasomal subunit Rpn6 is a molecular clamp holding the core and regulatory subcomplexes together Proc Natl Acad Sci U S A 2012 109 149 154 10.1073/pnas.1117648108 22187461 PMC3252951

[B37] Cajigas IJ Will T Schuman EM Protein homeostasis and synaptic plasticity EMBO J 2010 29 2746 2752 10.1038/emboj.2010.173 20717144 PMC2924649

[B38] Prigione A Fauler B Lurz R Lehrach H Adjaye J The senescence-related mitochondrial/oxidative stress pathway is repressed in human induced pluripotent stem cells Stem Cells 2010 28 721 733 10.1002/stem.404 20201066

[B39] Ebstein F Kloetzel PM Kruger E Seifert U Emerging roles of immunoproteasomes beyond MHC class I antigen processing Cell Mol Life Sci 2012 69 2543 2558 10.1007/s00018-012-0938-0 22382925 PMC11114860

[B40] Atkinson SP Collin J Irina N Anyfantis G Kyung BK Lako M Armstrong L A putative role for the immunoproteasome in the maintenance of pluripotency in human embryonic stem cells Stem Cells 2012 30 1373 1384 10.1002/stem.1113 22532526

[B41] Gaczynska M Goldberg AL Tanaka K Hendil KB Rock KL Proteasome subunits X and Y alter peptidase activities in opposite ways to the interferon-gamma-induced subunits LMP2 and LMP7 J Biol Chem 1996 271 17275 17280 10.1074/jbc.271.29.17275 8663318

[B42] Thomson JA Itskovitz-Eldor J Shapiro SS Waknitz MA Swiergiel JJ Marshall VS Jones JM Embryonic stem cell lines derived from human blastocysts Science 1998 282 1145 1147 9804556 10.1126/science.282.5391.1145

[B43] Ogawa K Nishinakamura R Iwamatsu Y Shimosato D Niwa H Synergistic action of Wnt and LIF in maintaining pluripotency of mouse ES cells Biochem Biophys Res Commun 2006 343 159 166 10.1016/j.bbrc.2006.02.127 16530170

[B44] James D Levine AJ Besser D Hemmati-Brivanlou A TGFβ/activin/nodal signaling is necessary for the maintenance of pluripotency in human embryonic stem cells Development 2005 132 1273 1282 10.1242/dev.01706 15703277

[B45] Greber B Lehrach H Adjaye J Control of early fate decisions in human ES cells by distinct states of TGFβ pathway activity Stem Cells Dev 2008 17 1065 1077 10.1089/scd.2008.0035 18393632

[B46] Hatakeyama S Ubiquitin-mediated regulation of JAK–STAT signaling in embryonic stem cells JAK-STAT 2012 1 168 175 10.4161/jkst.21560 24058766 PMC3670240

[B47] Miyazono K Ten DP Heldin CH TGF-beta signaling by Smad proteins Adv Immunol 2000 75 115 157 10879283 10.1016/s0065-2776(00)75003-6

[B48] Wang T The 26S proteasome system in the signaling pathways of TGF-beta superfamily Front Biosci 2003 8 d1109 d1127 10.2741/1057 12957830

